# Sustainable Lightweight Insulation Materials from Textile-Based Waste for the Automobile Industry

**DOI:** 10.3390/ma14051241

**Published:** 2021-03-05

**Authors:** Zengxiao Cai, Md Abdullah Al Faruque, Alper Kiziltas, Deborah Mielewski, Maryam Naebe

**Affiliations:** 1 Institute for Frontier Materials, Deakin University, Geelong, VIC 3216, Australia; z.cai@deakin.edu.au (Z.C.); malfaruq@deakin.edu.au (M.A.A.F.); 2 Research and Innovation Center, Ford Motor Company, Dearborn, MI 48121, USA; akizilt1@ford.com (A.K.); dmielews@ford.com (D.M.)

**Keywords:** wool, recycling, nonwoven, sound and thermal insulator, biodegradation, antimicrobial

## Abstract

Globally, automotive manufacturers are looking for ways to produce environmentally sustainable and recyclable materials for automobiles to meet new regulations and customer desires. To enable the needs for rapid response, this study investigated the feasibility of using waste and virgin wool fibres as cost-effective and sustainable alternatives for automotive sound and heat insulation using a chemical-free approach. Several properties of the currently available commercial automotive insulators were investigated in order to facilitate the designing of green wool-based needle-punched nonwoven materials. The effect of fibre diameter, nonwoven surface, layer structure, thickness, and area density on sound absorption and thermal resistance was investigated. The results suggested that the wool nonwoven materials, fabricated using waste and virgin wool fibres, possessed extremely efficient acoustic and thermal insulating properties comparable with the currently used commercial synthetic insulating materials. Besides, the wool nonwoven materials showed identical antibacterial and antifungal properties with a greater biodegradation rate (50%) than that of the commercial synthetic insulating materials. Hence, this study showed that natural wool fibres have the potential to be used as green, lightweight, and sustainable materials in the automobiles, while they qualify for Reuse–Recycle and Reuse–Recover purposes at the end-of-life of vehicles.

## 1. Introduction

In this modern era of environmental awareness, manufacturers and consumers are mainly focusing on the production and consumption of nature-based biodegradable, biocompatible and recyclable products [1,2,3,4]. The automobile industry is not exempt from this and seeks to fabricate various automobile parts derived from natural and renewable raw materials rather than traditional synthetic fibres (glass, carbon, aramid fibres etc.) and petroleum-based oils for polyurethane foams [5,6,7,8]. Besides, it has been found that every year, in the European Union, end-of-life vehicles (ELV) generate around 8–9 million tonnes of waste [9]. Moreover, as of 1 January 2015, the Directive 2000/53/EC on end-of-life vehicles set detailed quantified targets, which state that 85% of the vehicle must qualify for Reuse–Recycle and 95% for Reuse–Recover purpose [9]. This has pushed car manufacturers to consider the use of sustainable manufacturing materials such as natural fibres especially for the noise and heat insulation. The ability to isolate the mechanical noise, vibration, heat, and thermally insulate the interior of the vehicle leads to comfortable driving, and significant energy savings from the air-conditioning usage perspective. These insulation materials can be used for floor coverings, under the seat cover, door trim, pack panels, engine compartment, and on the ceiling [6,10]. Nonwovens, which can be easily made into different densities, thickness, and forms, have been applied in the car interior owing to their light weight, simple processing, flexibility, porosity, and sound insulation properties [11]. Needle-punching, a large-scale nonwoven manufacturing method using mechanical interlocking to bond, shows its advantages in simple processing and low cost rather than the other nonwoven web bonding methods. Fibres are interlocked during the needle-punching process, which substantially increases the resistance to the sound wave [12].

Natural fibres (flax, bamboo, kenaf, and jute) are increasingly being considered by the car manufacturers to offset the limitations of synthetic fibres [13,14]. However, their uses are limited to inner composite panels due to the harsh handle, poor aesthetic appearance, and limited insulating efficiency. The range of natural fibres, which can comprehensively satisfy the multiple functionalities (insulation, aesthetics, and comfort) of a car interior is therefore limited, and new opportunities have not been fully explored. Wool, as an animal protein fibre, is inherently flame retardant, odour resistant, aesthetically pleasing, luxuriously soft, and thermally and acoustically insulating [3,4,15]. This fibre possesses a very complex chemical and physical structure, which plays an important role in several functional properties and applications [3,15,16]. The surface of wool fibre is covered with overlapping cuticle cells (scales) separated by a cell membrane complex (CMC). The outermost external thin membrane named as the epicuticle consists of proteinaceous materials containing a high level of amino acid cysteine [17,18]. Due to its chemistry, wool can absorb water up to 30% of its weight without feeling wet, which may help in regulating the humidity of the car interior [3,19].

Although only a few reports have been published on the insulating properties of the wool fibres for their application in the building construction [20,21,22,23,24], the literature lacks information regarding both the acoustic and thermal characteristics of the nonwoven developed from wool as an automotive insulator. Ballagh et al. [25] studied the influence of wool fibre diameter, density, and flow resistivity on wool material acoustical properties. He found that the sound absorption coefficient of the wool material increased with the increasing density when the sample thickness was 25 mm. However, in the case of the thicker sample (100 mm thickness), the sound absorption coefficient increased with the density up to 47 kg/m^3^ and decreased with the further increment in the density [25]. Additionally, the sound absorption coefficient decreased with the increment in fibre diameter, but the wool acoustical property did not strongly rely on the flow resistivity. In another work, Broda and Bączek [26] investigated the influence of a multilayer structure on wool nonwovens on the sound absorption and found that the multilayer structure improved their sound insulating properties at a low number of layers.

There is limited information available on the performance of the commercially widely used insulating materials and their role in altering the insulating properties for the automotive interior. There are also very few, if any, reports on the application of only wool fibres (100%) as a candidate automotive insulator, although wool-based car interiors have been realised by some limousines for a long time. Therefore, this study focuses on two parts: First, it aims to understand the performance of current commercial automotive insulators associated with their construction and insulating properties. These results are important for estimating the required properties and insulation for designing an individualised car insulator from 100% natural wool fibres. Second, this study aims to utilise wool to address the combined limitations of synthetic and other natural fibres as an insulator. The fibre size and limitation of natural fibres was overcome by the use of a mixture of waste wool and virgin wool. A variety of densities and thicknesses of wool nonwoven material (WNM) was fabricated using the needle-punching method and systematically compared with the commercially available materials. The influence of fibre diameter, surface, layered structure, thickness, and area density on the acoustic properties were studied. Additionally, the thermal insulating properties, antibacterial and antifungal properties, and biodegradability of all the samples were investigated to better understand the suitability of the wool nonwoven materials (WNM) as renewable sound and thermal insulating materials to be used in different parts such as door trim, pack panels, engine compartment, and on the ceiling.

## 2. Materials and Methods

### 2.1. Materials

The virgin and waste wool fibres were supplied by the Commonwealth Scientific and Industrial Research Organisation (CSIRO), Geelong Waurn Ponds, Australia (collected from local farm supporting animal rights). Two types of commercially available insulating materials were provided by a local supplier. Due to the nature of the fibres (polyester (PET) and cotton/ polyester (CEL/PET)) used in the commercial insulating material, they were named PET and CEL/PET throughout this work. These insulating materials are usually used in door trim, pack panels, engine compartment, and on the ceiling to reduce noise and absorb heat.

### 2.2. Fabrication of Needle-Punched Wool Nonwoven Materials

The wool nonwoven materials (WNMs) were manufactured using the needle-punching method using a needle-punching machine (James Hunter, North Adams, MA, USA). In brief, waste and virgin wool fibres were mixed in a ratio of 1:1 and were then carded and parallel-laid to form a batt of an approximate area density (GSM) between 460 g/m^2^ and 790 g/m^2^. The needle-punching machine consists of 7128 needles on board. The working width of the nonwoven web was 1 m. Both the infeed and the draw-off speeds of the machine were 1.25 m/min, with a stroke frequency of 298 strokes/min. Different batts were needle-punched to different punch densities from 20 to 500 punches/cm^2^ to produce the WNM with altered thickness based on the work requirement. The layered structure of the WNM was covered with two layers of thin nonwoven polyester fabrics, (abbreviated to TNP, porous structure with a thickness of 0.25 mm and area density of 16.5 g/m^2^) on the needle-punched side or both sides of the WNM.

### 2.3. Thickness and Area Density

The thickness of the WNM and the commercial insulating materials (PET and CEL/PET) was measured using a digital thickness tester (M035A, UTS, Xiangcheng Region, Zhangzhou City of Fujian PR, China) with a load pressure of 4.14 KPa and a diameter of 28.7 mm. Three points on each sample were measured in a standard atmosphere (20 ± 2 °C and 62 ± 2% relative humidity) during the tests. Area density (GSM) was calculated based on Equation (1):

(1) $$\mathrm{Area}\;\mathrm{density}\;\left(g/m^{2}\right)\;=\;\mathrm{Weight}/\mathrm{Surface}\;\mathrm{area}$$

### 2.4. Morphology and Fibre Diameter Evaluation

The morphology of the commercial samples (PET and CEL/PET) and the WNM was analysed using a Jeol Neoscope SEM (Jeol Australasia Pty Ltd., Frenchs Forest, NSW, Australia). The samples were gold sputter-coated using Leica EM ACE600 (Leica Microsystems Pty Ltd., Macquarie Park, NSW, Australia) and then imaged under the Jeol Neoscope SEM (Jeol Australasia Pty Ltd., Frenchs Forest, NSW, Australia). The mean fibre diameter was measured using the image analysis software Image J (National Institutes of Health, Bethesda, MD, USA) and randomly calculated by selecting 100 fibres observed in the SEM image. Due to the intrinsic properties of wool fibres, its diameter was tested with OFDA 2000 (Optical-based Fibre Diameter Analyser, BSC Electronics, Ardross, Western Australia, Australia) using the optical image analysis. The wool fibres were cut into 2 mm short fibres. The 2 mm short wool fibres were scanned, measured and averaged. A minimum of 35,000 fibres was measured.

### 2.5. FTIR Analysis

To investigate the fibre type of the commercial insulating materials (PET and CEL/PET), the Fourier-transform infrared (FTIR) spectra analysis was accomplished under the Attenuated Total Reflectance (ATR) mode using Vertex 70 (Bruker Optik GmbH, Rudolf-Prank-Strabe, Ettlingen, Germany) spectrometer with a scan resolution of 4 cm^−1^ and 32 scans per sample between 400 and 4000 cm^−1^. Data were collected after baseline correction by OPUS 5.5 software (Bruker Optik GmbH, Rudolf-Prank-Strabe, Ettlingen, Germany).

### 2.6. Sound Absorption Evaluation

The acoustic properties of all the materials (PET, CEL/PET, and WNM) were characterised using a sound Impedance Tube (CSIRO, Victoria, Australia,) according to the ASTM E1050-98 [27] by measuring the sound absorption coefficient (α), as shown in Equation (2):

(2) $$\alpha =\;\frac{4}{\left[n+\left(\frac{1}{n}\right)+2\right]}$$

where α is the sound absorption coefficient and n is the ratio of the intensity of the sound waves generated by the source and the intensity of the reflected sound waves.

**Figure 1:**
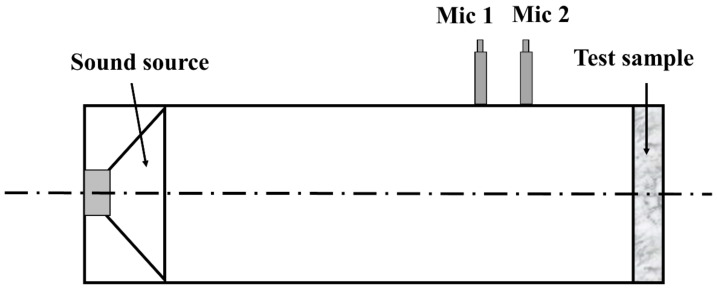
A schematic diagram of the Impedance Tube.

Briefly, samples were cut into two different sizes (34 and 78 mm) using a sample cutter (TFT2311 Uigeurno torielli, Australia). The 34 mm tube was used to test the sound absorption coefficient at a (low) frequency between 108 and 2390 Hz. The sound absorption coefficient at a (high) frequency from 2002 to 5889 Hz was tested using the 78 mm tube. The calibration was performed before conducting the test. Samples were mounted into the sample holder, which was clamped onto the tube for testing (Figure 1). For each material, three samples were tested in each tube size to cover the whole frequency ranged between 108 and 5889 Hz. The noise reduction coefficient (NRC) of all the materials were calculated according to Equation (3) [28].

(3) $$\mathrm{NRC}=\;\frac{\left(\alpha 250\;\mathrm{Hz}\;+\;\alpha 500\;\mathrm{Hz}\;+\;\alpha 1000\;\mathrm{Hz}\;+\;\alpha 2000\;\mathrm{Hz}\right)}{4}$$

NRC, the percentage of sound absorbed by the material (the percentage of the sound that is not reflected into the room) is an average of sound absorption coefficient (*α*) at four frequencies of 250, 500, 1000, and 2000 Hz [28]. In this study, similar frequencies (247.6, 495.3, 1001.3, and 2002.6 Hz) were used to calculate the NRC.

### 2.7. Thermal Resistance

The thermal resistance measurement of all the samples (PET, CEL/PET, and WNM) was carried out using a Sweating Guarded Hotplate/Espec Chamber (Measurement Technology Northwest, Seattle, WA, USA) according to the ASTM F1868 (part C) [29]. Three samples with a dimension of 50 cm × 50 cm were prepared from each area density of the nonwoven wool and commercial samples. The temperatures of the test plate and ambiance were maintained at 35 ± 0.1 °C and 20 ± 0.1 °C, respectively. The air velocity was set to 1 ± 0.1 m/s. The relative humidity of the testing chamber was adjusted at 65 ± 4%. The average of three measurements was reported as the thermal resistance for each material.

### 2.8. Biodegradability

The biodegradability of the commercially available insulating materials (PET and CEL/PET) and the WNM was evaluated using Australian standard low-density soil (AS 4419, Standards Australia International Ltd., Sydney, NSW, Australia) [30]. All the samples were cut into a circular shape and buried into the soil in a container. The container was kept inside an incubator at 30 °C for six months. Every seven days, water was sprayed into the containers, due to the water evaporation from the container (not airtight). Samples were carefully taken out, washed, dried and weighed every month to calculate their weight loss (%). Dried samples were also scanned before and after degradation using Jeol Neoscope SEM (Jeol Australasia Pty Ltd., Frenchs Forest, NSW, Australia). A Leica EM ACE600 gold coater (Leica Microsystems Pty Ltd., Macquarie Park, NSW, Australia) was used to coat fibres before imaging. The *weight loss* (%) of the samples was calculated by following Equation (4) [31].

(4) $$Weight\;loss\;\left(\%\right)\;=\;\frac{Wo-Wt}{Wo}\;\times \;100$$

where *Wo* and *Wt* represent the initial weight and the final weight of the samples at a given time (*t*), respectively.

### 2.9. Antibacterial and Antifungal Test

Antibacterial properties of PET, CEL/PET and WNM were evaluated according to the method SN 195920. The commercial insulating materials (PET and CEL/PET) and the WNM were cut into 3 cm diameter discs and placed in the centre of a tryptic soy agar plates and inoculated with a cell suspension (10^5^ CFU/mL) of *Escherichia coli* (*E.coli*; ATCC no. 11229). The plates were then incubated for 24 h at 37 °C and then assessed for bacterial growth.

Antifungal properties of PET, CEL/PET, and WNM were assessed according to the AATCC TM030-2017. All the samples were cut into 3 cm diameter discs. Potato Dextrose Agar (Sigma-Aldrich, Sydney, Australia) was used as the test media. *Penicillium varians* (also known as *Penicillium funiculosum*, ATCC 10509) with a spore population of 1.0 × 10^6^ CFU/mL were used as inoculum and were distributed (0.5 ± 0.1 mL) evenly over the surface of the agar. Pre-wet specimens were autoclaved (The Chipmunk, Atherton, Australia) and placed on the agar surface. The inoculum (0.2 ± 0.01 mL) was evenly distributed over each disc using a sterile pipette (Interpath, Heidelberg West, Australia). *Penicillium varians* growth on these three different materials was observed after 2, 7, and 14 days of culture at a temperature of 28 ± 1 °C.

### 2.10. Statistical Analysis

All experiment data were shown as mean ± standard deviation. Statistical differences were analysed by Two-Sample *t*-Test using statistical software in the Origin 2019 (OriginLab Corporation, Northampton, MA, USA). The differences with *p* < 0.05 were considered significantly different.

## 3. Results

### 3.1. Preliminary Studies on the Performance of Commercial Automotive Insulators

**Figure 2:**
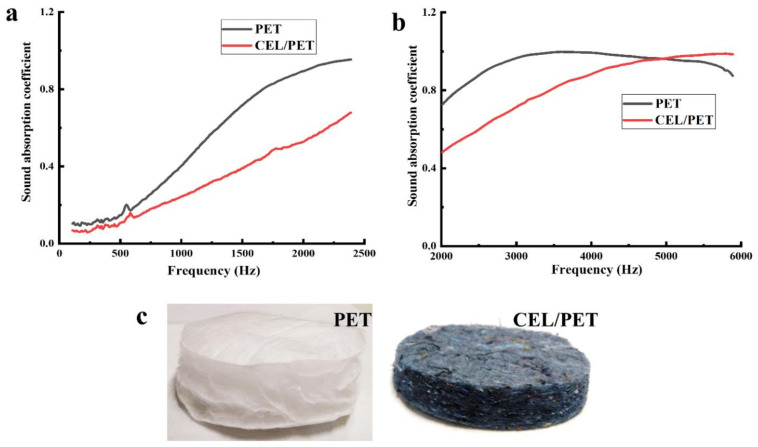
Two commercial insulating materials: (**a**) sound absorption coefficient in the low-frequency range, (**b**) sound absorption coefficient in the high-frequency range and (**c**) digital photos of PET and CEL/PET.

The sound absorption coefficient (α), noise reduction coefficient (NRC), area density (GSM), and the thickness of the commercial insulating materials (PET and CEL/PET) are summarised in Figure 2 and Table 1, respectively. While the CEL/PET illustrated a more compact structure (Figure 2c) and twice the area density (750 GSM) than PET (350 GSM), it showed a much lower sound absorption coefficient than PET (Figure 2a,b). However, PET was thicker (24 mm) than CEL/PET, which could be the reason for its higher sound absorption coefficient. Although it has been reported that both the area density and thickness contributed to the sound absorption properties [32,33], it can be seen that the material thickness of both PET and CEL/PET played an important role to improve the acoustic properties. However, it is worth mentioning that due to the differences in the fibre types of these two commercial insulating materials, the sound insulation properties are different, which is discussed in the following section.

### 3.2. Evaluation of the Fibre Diameter and Its Influence on the Sound Absorption

**Figure 3:**
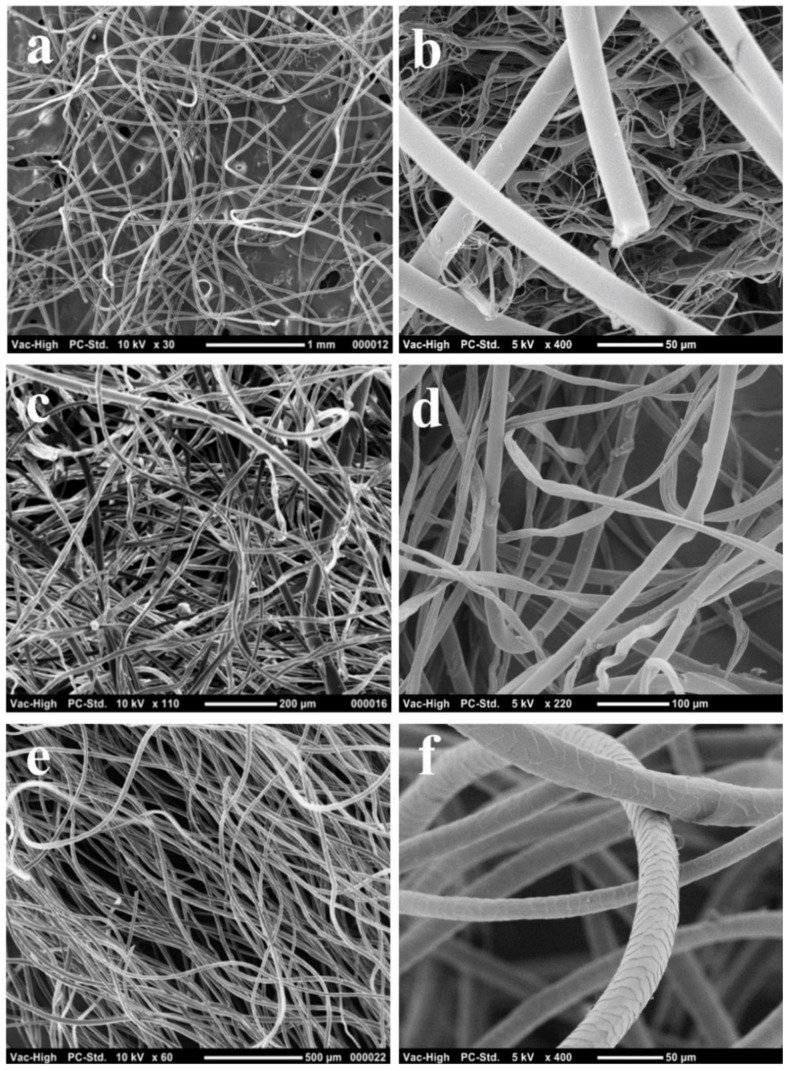
Scanning electron microscopy (SEM) images of fibres in different insulating materials: (**a**) low magnification of PET fibres, (**b**) high magnification of PET fibres; (**c**) low magnification of CEL/PET fibres, (**d**) high magnification of CEL/PET fibres; (**e**) low magnification of wool fibres; and (**f**) high magnification of wool fibres.

Fibre morphology and diameter of PET, CEL/PET, and wool samples were investigated and are shown in Figure 3 and Table 2, respectively. The low and high magnification SEM images of PET fibres (Figure 3a,b) showed a mixture of both coarser (mean diameter of 19.5 μm) and finer (mean diameter of 2 μm) fibres. While traces of yarns mixed with the fibres were observed in the low and high magnification SEM images of CEL/PET (Figure 3c,d), the samples showed coarser and finer fibres with a mean diameter of 15.5 and 10 μm, respectively. The SEM images of PET showed fibres with the uniform, round, and smooth continuous surfaces, indicating these are possibly made of the synthetic fibres. However, a combination of uniform and smooth surfaces (synthetic fibres) and flat, twisted, ribbon-like surfaces were observed in the CEL/PET samples (Figure 3d). This criterion specified the presence of a blend of cotton and synthetic fibres in the CEL/PET insulating materials. In the case of the WNM, overlapping scales were observed on the fibres (Figure 3f).

**Figure 4:**
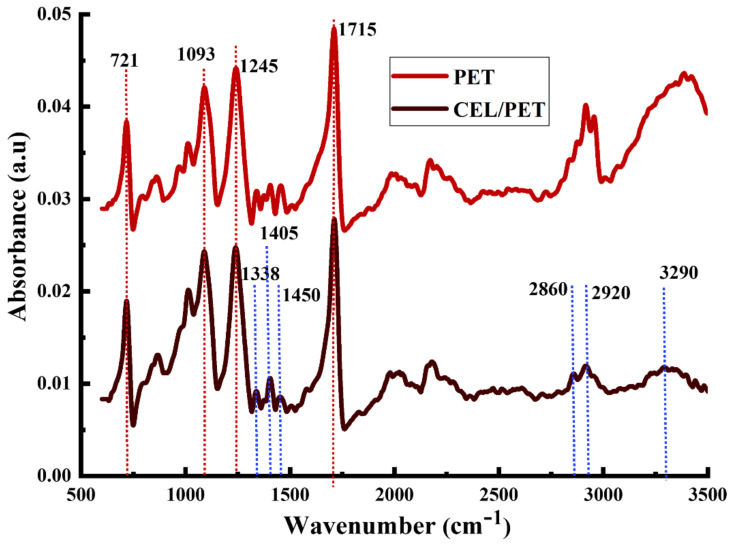
Fourier-transform infrared (FTIR) spectrum of the commercial insulating materials.

To further investigate the type of fibres used in the commercial samples (PET and CEL/PET), FTIR analysis was carried out and the spectra are shown in Figure 4. Both the PET and CEL/PET possess the identical peaks at 721, 1093, 1245, and 1715 cm^−1^, which represents C–H aromatic ring wagging, C–O stretching, ester C–O stretching, and C=O stretching, respectively those are directly expressing the presence of Polyethylene terephthalate (polyester) in their chemical structure [34,35]. Besides, the peaks positioned at 2900 and 3400 cm^−1^ are representing the vibration of the C–H stretching and O–H stretching, respectively, might be representing the existence of PET [36,37]. On the other hand, the CEL/PET insulating material showed specific peaks at 1338 cm^−1^ (–CH bending), 1405 and 1450 cm^−1^ (–OH bending), 2860 and 2920 cm^−1^ (–CH stretching), and 3290 cm^−1^ (stretching of –OH groups) those are related to the fibres with cellulosic structure [34,35] and more specifically cotton fibres (as confirmed by SEM). Therefore, from both the SEM and FTIR analysis, it can be claimed that the commercial insulating materials, PET and CEL/PET, were composed of polyester fibres and a blend of cotton/polyester fibres, respectively.

From Table 2, it is evident that the PET materials made of synthetic fibres (polyester as confirmed by FTIR) possessed a range of very fine fibres (2 μm) compared to the CEL/PET and WNM. Having fine fibres in the same area density of the nonwoven materials, indicated the presence of a higher amount of fibres compared to the nonwoven materials made of coarser fibres [12]. This results in higher air resistance and sound absorption properties of the nonwoven materials, as the fine fibres produce more airflow resistance due to the higher friction of sound waves [12,38]. Additionally, with the same area density, finer fibres have a higher fibre quantity compared to the coarser fibres, and this propensity increases the tortuous path and airflow resistance [39], which in turn increases the sound absorption property. Moreover, the use of finer fibres might assist in reducing the area density of the fabricated nonwovens, which also helps to address the heavyweight of the nonwoven insulating materials. Therefore, in this study, the finer virgin wool fibres (12 μm) were mixed with the coarser waste wool fibres (28 μm) in a ratio of 1:1 to fabricate the wool nonwoven materials (WNM) to investigate the acoustic properties.

### 3.3. Influence of the Needle-Punched Surface on the Sound Absorption of the WNM

**Figure 5:**
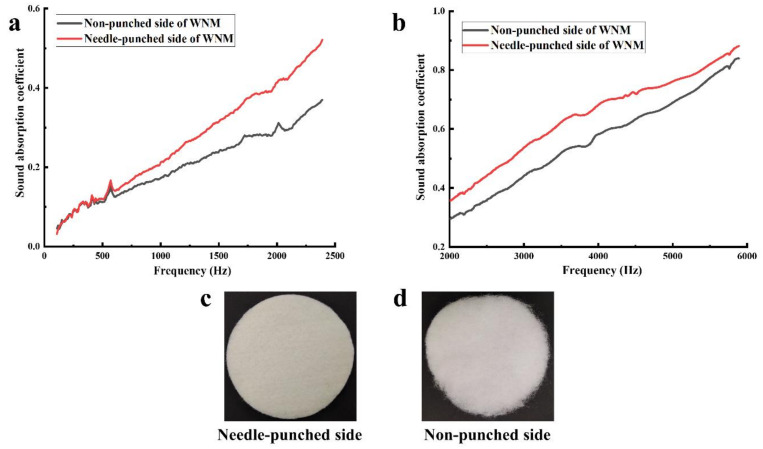
The surface of the wool nonwoven material (WNM): (**a**) sound absorption coefficient in the low-frequency range, (**b**) sound absorption coefficient in the high-frequency range, (**c**) digital image of the needle-punched side, and (**d**) digital image of the non-punched side.

The WNM was fabricated by the needle-punching mechanism that created two different surface structures on either side of the WNM. The needle-punched side showed a compact plain and porous surface (Figure 5c), whereas the non-punched side with a loose rough fibre surface (Figure 5d). Figure 5a,b demonstrated the sound absorption coefficient of the WNM measured on two sides in the low and high-frequency range, respectively. The needle-punched side showed a higher sound absorption coefficient in all the frequencies ranged between 108 and 5889 Hz. In addition, this side of the WNM showed higher NRC (0.29) compared to the non-punched side (0.17). The higher sound absorption coefficient and NRC of the needle-punched side could be attributed to the compact but porous surface of this side. It is likely that when the sound wave strikes a relatively porous surface, a lower proportion of the sound will be reflected compared to the more open surface [40]. The reason might be due to the fact that the porous surface allows many internal reflections, resulting in more absorption and less reflection [40,41]. Shahani et al. [41] investigated three different surface acoustic properties of needle-punched nonwoven fabrics and found the plain surface showed the highest NRC rather than the velour and cord surfaces due to its compact structure, porosity, and fibrous assembly. Therefore, in further investigations of the WNM, the plain surface of the needle-punched WNM was fitted to face the Impedance Tube sound source in the following sections.

### 3.4. Influence of Layered Structure on the Sound Absorption of the WNM

**Figure 6:**
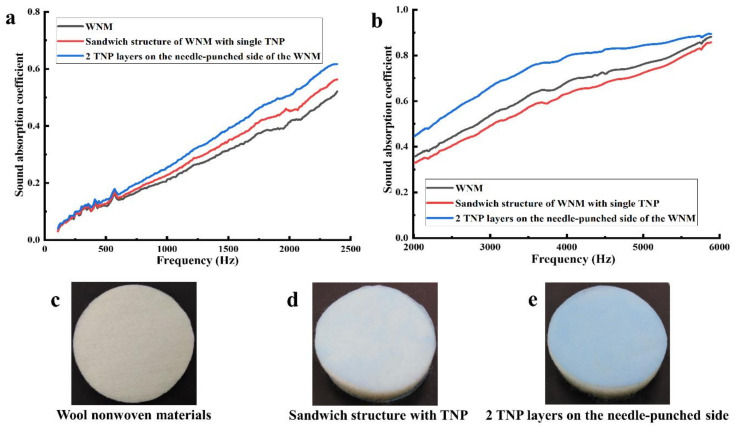
Properties of the wool nonwoven materials with and without thin nonwoven polyester (TNP): (**a**) sound absorption coefficient in the low-frequency range, (**b**) sound absorption coefficient in the high-frequency range (**c**) wool nonwoven materials, (**d**) sandwich structure of the WNM with single layer TNP, and (**e**) two TNP layers on the needle-punched side of the WNM.

The sound absorption tendency of the porous materials is greatly improved with the addition of a multilayer structure with the insulating materials [42,43]. Therefore, in this study, we profoundly investigated the sound absorption of the multi-layered structure WNM by simply adding a single layer of thin nonwoven polyester (TNP, porous structure with a thickness of 0.25 mm and GSM of 16.5 g/m^2^) on two sides of the WNM (sandwich structure, Figure 6d). Also, we added two layers of TNP on the needle-punched side of the WNM (Figure 6e). As shown in Figure 6a,b and Table 3, the sound absorption coefficient improved with the addition of the TNP layers. However, the WNM containing the two TNP layers on the needle-punched side (Figure 6e) showed higher sound absorption properties than the sandwich structure with a single layer of TNP on each side (Figure 6d). This might be because the two TNP layers enhanced the compact structure of WNM on the needle-punched side by adding extra layers of barriers in front of the sound source of the Impedance Tube. Additionally, the porous structure of the TNP layer assisted in sound absorption. The NRC (Table 3) of the WNM with the TNP layers (0.25) on the needle-punched side was comparable with the commercial insulating material, CEL/PET (0.24), where the WNM was almost 1.5 times lighter and 1.5 times thicker than the CEL/PET (747 GSM and 12.9 mm, respectively).

### 3.5. Effect of Thickness on the Sound Absorption of the WNM

**Figure 7:**
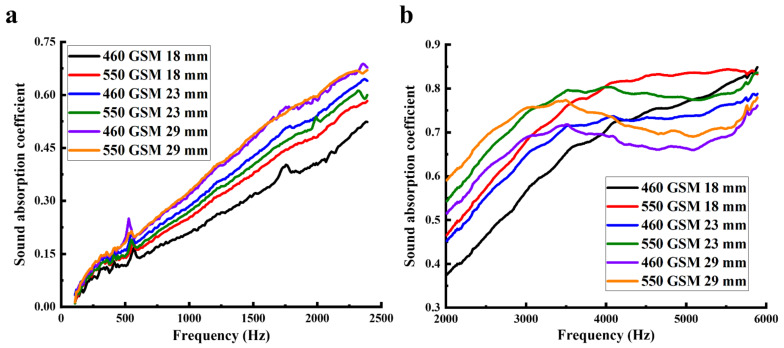
Sound absorption properties of the wool nonwoven materials of 460 and 550 GSM with different thickness: (**a**) sound absorption coefficient in the low-frequency range and (**b**) sound absorption coefficient in the high-frequency range.

The effect of thickness on the acoustic properties of the WNM was investigated by fabricating a range of WNM with different thicknesses ranging from 18 to 29 mm, which were needle-punched into two different area densities of 460 and 550 GSM. The sound absorption coefficient of the fabricated WNM was evaluated in both low (Figure 7a) and high-frequency ranges (Figure 7b). As shown in Figure 7a, at the low-frequency area and for both area densities of 460 and 550 GSM, the wool nonwoven materials (WNMs) with the higher thickness resulted in better sound absorption coefficient compared with the lower thickness. However, the differences between the thicker and thinner WNM in the sound absorption coefficient at the high-frequency range (2002–5889 Hz, Figure 7b) was not as significant as its effect on the sound absorption coefficient within the low-frequency range. For the WNM of 460 GSM in the range of 2002–3500 Hz, the WNM with the greater thickness contributed to the higher sound absorption coefficient. However, after 3500 Hz, the WNM with lower thickness induced a higher sound absorption coefficient. A similar trend was evident on the WNM with an area density of 550 GSM. Therefore, it can be concluded that thickness has a direct relationship with sound absorption at lower frequencies but insignificant influence at higher frequencies [44]. Thus, WNM can be used in different thickness range based on the sound insulating requirements of different parts of the automobile for different frequency ranges.

### 3.6. Effect of the Area Density on Sound Absorption of the WNM

**Figure 8:**
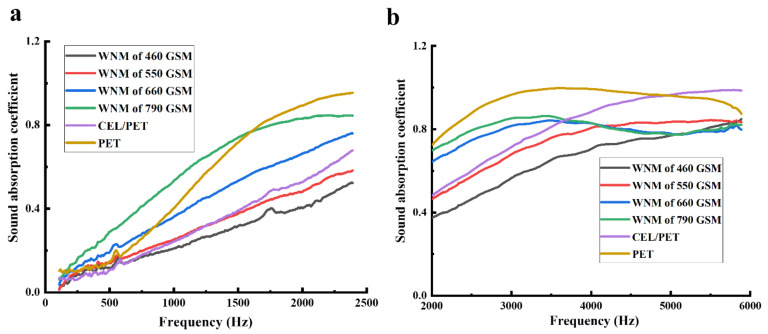

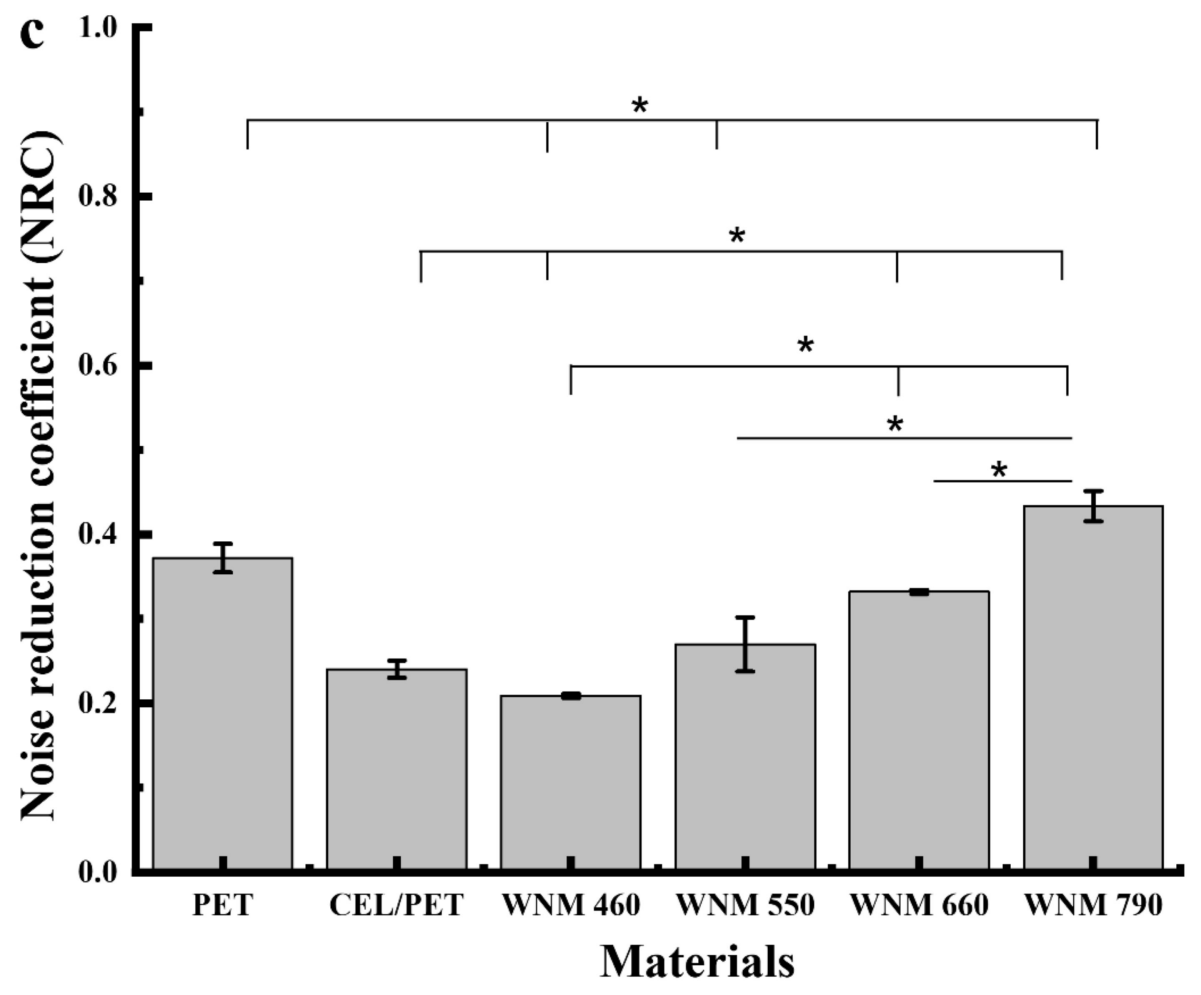
Sound insulating properties of the WNMs with different area density and commercial materials (PET and CEL/PET): (**a**) sound absorption coefficient of all the materials in the low-frequency range, (**b**) sound absorption coefficient of all the materials in the high-frequency range, and (**c**) noise reduction coefficient (NRC) of all the materials. Error bars show the standard deviations—statistical significance: *p* < 0.05 (*).

Wool nonwoven materials (WNMs) with different area densities of 460, 550, 660, and 790 GSM were fabricated to further investigate the influence of area density on the sound absorption properties. Table 4 shows the thickness and area density of the fabricated WNM compared with the commercial materials (PET and CEL/PET). The acoustic properties of the fabricated WNM and the commercially available PET and CEL/PET materials are summarised in Figure 8. The WNM with the area density of 790 GSM showed the highest sound absorption coefficient (Figure 8a,b) compared with the other three WNM (460, 550, and 660 GSM), especially in the lower frequency range (108–2390 Hz). It also showed a higher sound absorption coefficient compared to the PET in a range of frequency up to 1600 Hz. The WNM of 460 GSM showed the lowest sound absorption coefficient from 108 to 5050 Hz. In the frequency range from 108 to 4091 Hz, the sound absorption coefficient of the WNM increased with the increase of the area density. As mentioned earlier, the area density of the WNM refers to the number of fibres in a certain area, and higher area density indicates the presence of higher proportion of fibres in a specific area, which induces more resistance to the sound wave [26,45] and contributes to the higher sound absorption tendency [46,47,48]. As shown in Figure 8c, the WNM with an area density of 790 GSM showed the highest NRC (0.43), significantly different (*p* < 0.05) from the other WNM and the commercial materials (PET and CEL/PET). The WNM of 660 GSM showed slightly lower NRC (0.33) than that of PET (0.39) but significantly higher than the CEL/PET (0.24), (*p* > 0.05). Given that lightweight materials for car interiors possess advantages due to their higher energy savings [49,50], we aimed to fabricate the WNM with the lower area density, when higher thickness possibly would compensate for the effect of area density on the noise reduction. Therefore, as discussed, all the fabricated WNM except 790 GSM were fabricated with higher thickness and lower area density than CEL/PET, which showed optimum results. The result also can be compared with the Broda and Bączek’s [26] work, when the nonwoven wool with higher area density (~920 GSM) showed lower NRC (0.37) than our fabricated 790 GSM with significantly higher NRC (0.43).

### 3.7. Thermal Resistance Evaluation

**Figure 9:**
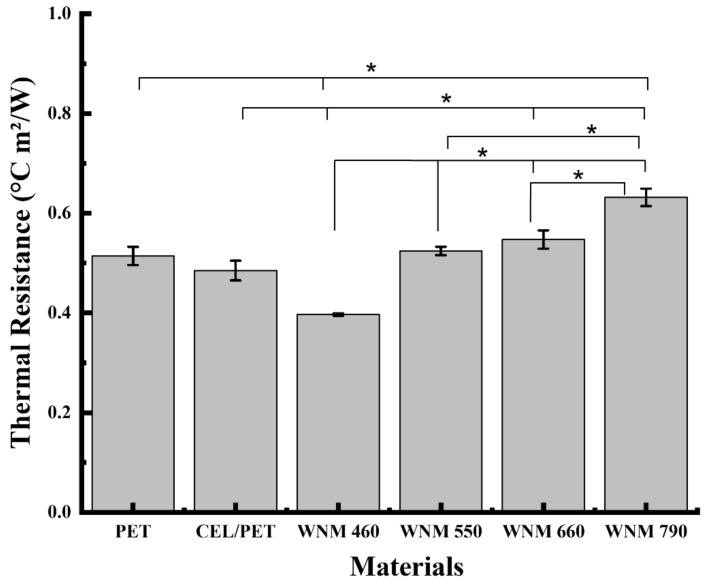
Thermal resistance of the commercial insulating materials (PET and CEL/PET) and the wool nonwoven materials (WNMs) with different area density. Error bars show the standard deviations—statistical significance: *p* < 0.05 (*).

Thermal resistance is a heat property and a measurement of a temperature difference by which an object or material resists a heat flow. Intrinsic thermal resistance (Rcf), [(°C m²/W)], the resistance to dry heat transfer provided by the textile system alone were evaluated for the fabricated WNM and the commercial insulating materials (PET and CEL/PET) (Figure 9). As shown in Figure 9, thermal resistance increased with enhancing the area density of the WNM. This might be due to the wool crimp—the natural waviness of the wool fibre. The higher proportion of wool fibres led to the higher scattering of the wool fibres in the nonwoven structure and trapping more air inside the natural crimps, influencing the thermal resistance of the wool nonwoven materials (WNMs).

The WNM with an area density of 790 GSM showed significantly higher thermal resistance (0.63 °C m^2^/W) than the other WNMs and commercial insulating materials (PET and CEL/PET), which eventually demonstrated the lowest heat loss and highest thermal insulating properties. The WNM of 660 GSM with higher area density but similar thickness to the PET sample showed higher thermal resistance (0.54 °C m^2^/W) to the PET (0.51 °C m^2^/W). This can also be potentially attributed to the PET fine fibres (observed by SEM) and inherent thermal properties of polyester. The WNM of 550 and 660 GSM with lower area density but higher thickness than CEL/PET, showed higher thermal resistance than the CEL/PET (0.49 °C m^2^/W). As observed by the SEM images and FTIR spectra, the CEL/PET contains cotton fibres in its structure. Compared to the wool fibres, the cotton fibres are thermally conductive, which may contribute to the thermal resistance properties of the CEL/PET. Although the thickness and the area densities are the significant parameters affecting the thermal resistance of the materials [51,52], in developing insulating materials, the effect of fibre types and diameter should not be neglected.

**Figure 10:**
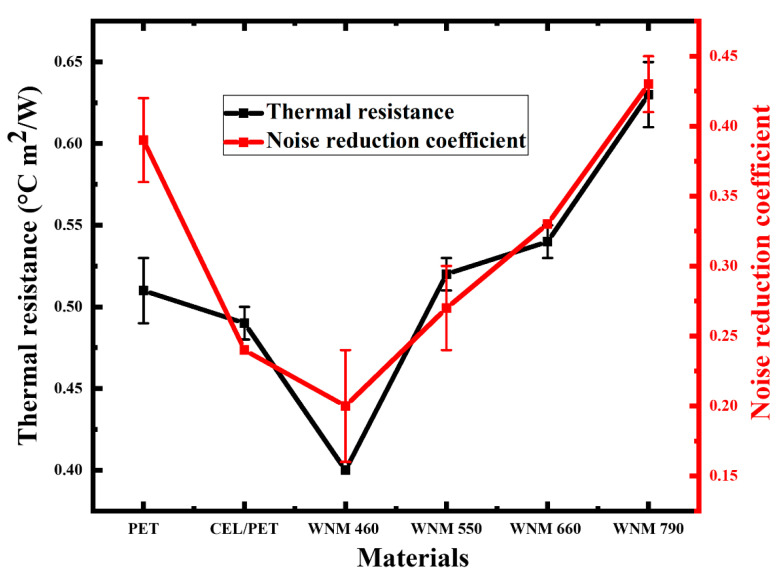
Thermal resistance and noise reduction coefficient of PET, CEL/PET, and WNM.

Figure 10 shows the relationship between the thermal resistance and the noise reduction coefficient of the commercial insulating materials and the WNM. Thermal resistance and noise reduction coefficient showed a relatively similar pattern towards each sample, which means for individual fibre type these two insulating properties are dependent on similar parameters such as fibre diameter, thickness, and area density [48,53].

### 3.8. Analysis of the Antibacterial and Antifungal Properties

**Figure 11:**
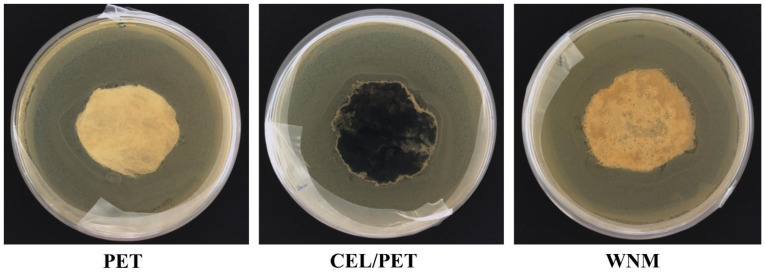
Antibacterial test of PET, CEL/PET, and wool nonwoven materials (WNMs) with *E. coli* after 24 h incubation.

**Figure 12:**
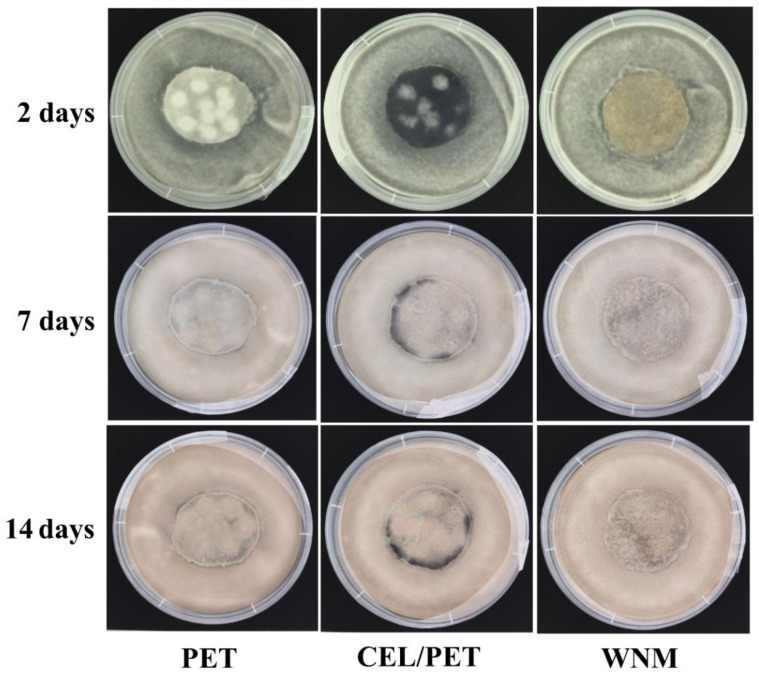
Antifungal test of PET, CEL/PET, and wool nonwoven materials (WNM) with *Penicillium varians* after 2, 7 and 14 days incubation.

Wool as a natural fibre is made of animal protein, which might be much more favourable for the growth of bacterial and fungi compared to synthetic materials. Therefore, we investigated the antibacterial and antifungal properties of the commercial insulating material (PET and CEL/PET) and the WNM to further understand the practical application of the WNM as the automotive interior. Representative images of antibacterial activity of PET, CEL/PET, and WNM are shown in Figure 11, where *E. coli* growth can be seen on all of the samples. Interestingly, some minor clear areas around the WNM can still be seen, which indicated some minor antibacterial activity of the WNM. The antifungal property was tested after 2, 7 and 14 days culture with *Penicillium varians* (Figure 12). There were much fewer fungi growing on the WNM compared to the PET and CEL/PET after two days of incubation. However, *Penicillium varians* grew on all these three types of materials, and there was no significant difference among them after 7 and 14 days incubation. This confirmed that natural animal protein wool fibre showed similar antibacterial and antifungal properties to the commercially available insulating materials (PET and CEL/PET) used in the automotive industry.

### 3.9. Biodegradation

**Figure 13:**
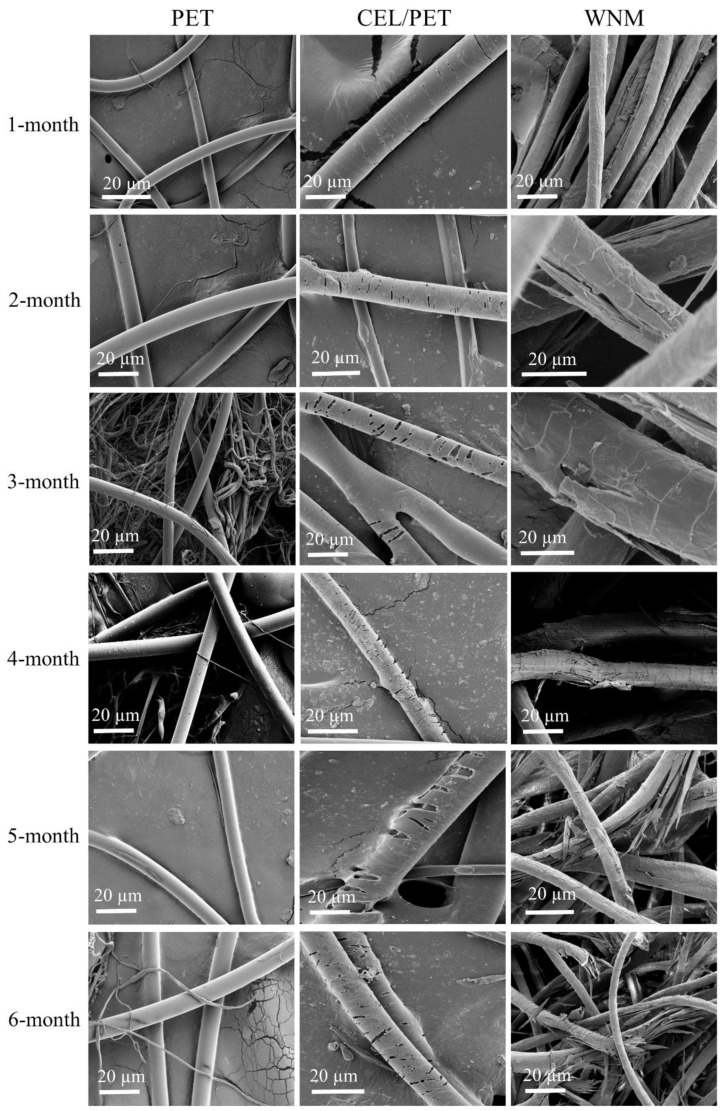
SEM images of the PET (Left), CEL/PET (Middle), and WNM (Right) after the soil biodegradation within 6-months.

**Figure 14:**
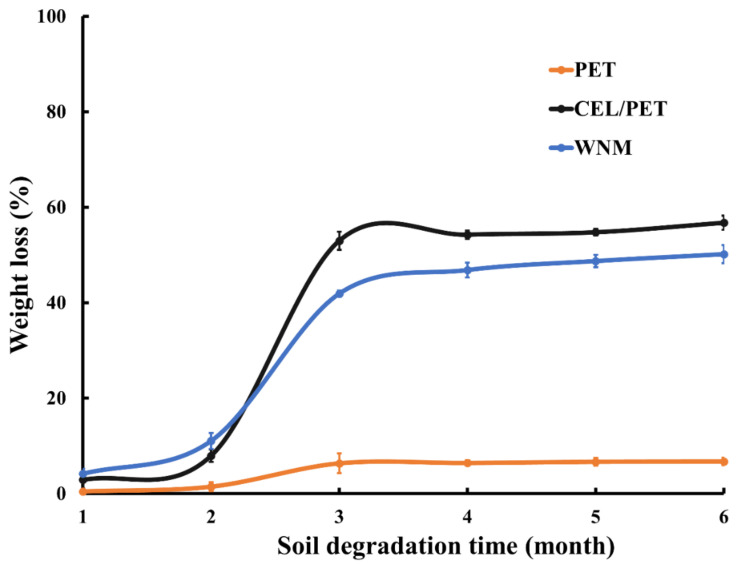
Month-wise weight loss (%) of the PET, CEL/PET, and WNM.

To evaluate if the materials used in this study would qualify for Reuse-Recycle and Reuse-Recover at the end-of-life vehicles, the biodegradability of commercially insulating materials and the fabricated WNM samples was tested. The soil biodegradation of the PET, CEL/PET, and wool nonwoven materials (WNMs) was evaluated based on their weight loss (%) [31]. Figure 13 demonstrates the SEM images of the PET, CEL/PET, and WNMs during six months of evaluation. Figure 14 shows the trend of weight loss (%) for six months. The biodegradability of the natural fibres is higher than most of the synthetic fibres [31]. Similar behaviour was found for the PET, CEL/PET, and WNMs. As the PET was composed of purely synthetic materials (polyester as shown by FTIR spectra), its degradation rate was very low and negligible (~7%). On the other hand, both the CEL/PET and the WNMs showed more than 50% soil degradation in the period of six month. Though it was expected that, due to the presence of polyester, weight loss (%) of the CEL/PET was lower that WNM, it was marginally higher than that of the WNMs (Figure 14), which might be due to the presence of cotton fibre in CEL/PET.

Although the cotton fibre possessed around 70% crystalline region and only 30% amorphous region, its rate of soil degradation is high, as the crystalline parts are arranged towards the fibre axis [54,55]. On the contrary, wool fibres are composed of different amino acids, highly sulphur cross-linked keratin protein, and a tough fibrous surface with water-repelling membranes [55,56]. This might be the reason that wool fibres are more resistant to various moths, insects, and microorganisms and result in lower soil degradation compared with cotton fibres [55]. Therefore, the WNMs showed a lower soil degradation compared to the CEL/PET, although its biodegradability was much higher (more than seven times) than the synthetic PET insulating material.

## 4. Conclusions

One of the most important challenges for the automotive industry is noise reduction. Drivers and passengers expect a quiet acoustic environment and seek better noise, vibration, and harshness (NVH) environments. Previous attempts to reduce the noise inside of vehicles typically meant adding more material and weight, using synthetic materials. However, this work uses lightweight, green, environmentally friendly, and biodegradable wool fibres as the insulating material to create a peaceful, noise-free environment. In this study, the acoustic properties of the commercially available insulating materials (PET and CEL/PET) were investigated. The effect of the area density, thickness, fibre types, and fibre diameters on the sound absorption coefficient was studied as a reference for fabricating wool nonwoven materials (WNM) from the waste and virgin wool fibres. The fabricated WNM using the needle-punching method showed comparable acoustic properties and thermal resistance with the commercial insulating material (PET and CEL/PET). It has been found that fibre diameter, surface of the nonwoven materials, multilayer structure, thickness and area density played an important role in the sound absorption properties. Besides, the fabricated WNM with different thickness (ranged between 18 and 29 mm) and area density (ranged from 460 to 790 GSM) showed an excellent sound absorption phenomenon in the altered sound frequency range. Therefore, it is possible to fabricate the WNM with different thickness for various parts of the automobile for the sound insulating purpose. The thermal property of the WNM showed a similar trend to the acoustic property with different parameters such as fibre diameter, thickness, and area density. WNMs exhibited identical antibacterial and antifungal properties as of the commercial insulating materials. The biodegradability of the WNM (50%) was more than seven times higher compared to the synthetic PET (7%), pointing to the fact that the fabricated WNM will significantly reduce the waste at the end-of-life vehicles.

## Figures and Tables

**Table 1:** Noise reduction coefficient (NRC), area density (GSM), and thickness (± standard deviation) of PET and CEL/PET.

Materials	NRC	GSM (g/m^2^)	Thickness (mm)
PET	0.394 ± 0.027	350 ± 31	23.7 ± 0.5
CEL/PET	0.241 ± 0.003	747 ± 40	12.9 ± 0.5

**Table 2:** Mean fibre diameter (± standard deviation) of materials studied in this work.

Fibre	PET Coarse Fibre	PET Fine Fibre	CEL/PET Coarse Fibre	CEL/PET Fine Fibre	Waste Wool	Virgin Wool
Diameter (μm)	19.4 ± 3.9 *	2.2 ± 1.5 *	15.3 ± 4.5 *	10.2 ± 3.7 *	28.0 ± 2.0 **	11.9 ± 2.6 *

* data collected from SEM. ** data collected from OFDA.

**Table 3:** Thickness, area density (GSM), and noise reduction coefficient (NRC) of various WNM (± standard deviation).

Materials	Thickness (mm)	GSM (g/m^2^)	NRC
WNM	18.6 ± 0.5	462 ± 2	0.209 ± 0.006
Sandwich structure with 1-layered TNP	19.1 ± 0.5	492 ± 1	0.224 ± 0.002
2 TNP layers on the needle-punched side	19.1 ± 0.5	492 ± 1	0.249 ± 0.007

**Table 4:** Area density and thickness of PET, CEL/PET, and different WNMs.

Materials	GSM (g/m^2^)	Thickness (mm)
PET	350 ± 31	23.7 ± 0.5
CEL/PET	747 ± 40	12.9 ± 0.5
WNM of 460 GSM	462 ± 2	18.6 ± 0.5
WNM of 550 GSM	546 ± 4	18 ± 0.0
WNM of 660 GSM	659 ± 2	25.3 ± 0.8
WNM of 790 GSM	783 ± 17	31.7 ± 0.4

## Data Availability

The raw/processed data required to reproduce these findings cannot be shared at this time as the data also forms part of an ongoing study.
